# Wearable technology identifies differences in change of direction kinetics and kinematics in soccer players with a history of anterior cruciate ligament reconstruction

**DOI:** 10.1002/ksa.12679

**Published:** 2025-04-15

**Authors:** Joao Belleboni Marques, Vasileios Sideris, Rodney Whiteley, Paul James Read, Matheus Machado Gomes, Paulo Roberto Pereira Santiago

**Affiliations:** ^1^ Aspetar, Orthopedic and Sports Medicine Hospital, FIFA Medical Center Riadh Assessment and Movement Analysis Lab Doha Qatar; ^2^ Faculty of Medicine, Graduate Program in Rehabilitation and Functional Performance University of Sao Paulo (USP) Ribeirao Preto Sao Paulo Brazil; ^3^ Department of Physical Education and Sport Science University of Thessaly Trikala Greece; ^4^ Faculty of Sport, Allied Health and Performance Science St Mary's University London UK; ^5^ School of Sport and Exercise University of Gloucestershire Gloucester UK; ^6^ Division of Surgery and Interventional Science University College London London UK; ^7^ School of Physical Education and Sport of Ribeirao Preto, Biomechanics and Motor Control Lab (LaBioCoM) University of Sao Paulo (USP) Ribeirao Preto Sao Paulo Brazil

**Keywords:** knee biomechanics, side‐step cutting, soccer, ACL reconstruction, wearable technology

## Abstract

**Purpose:**

This study investigates change of direction (COD) performance and biomechanics using wearable technology in athletes with a history of anterior cruciate ligament reconstruction (ACL‐R) compared to healthy controls.

**Methods:**

A within and between subjects' cross‐sectional design was used. The sagittal plane kinematics of the hip, knee, and ankle during 90° side‐step cutting were measured with inertial measurement units, while the vertical force was recorded with insoles in the players' boots. Twenty‐six professional soccer players participated (mean age 22.7 ± 3.7 years, height 177.8 ± 5.1 cm, weight 69.4 ± 8.5 kg). Sixteen players were healthy controls, and 10 were in a full‐time ACL‐R rehabilitation programme, assessed 9 months post‐surgery. Mixed model analysis and statistical parametric mapping were used to compare COD completion time, kinetics, and kinematics between limbs (involved vs. uninvolved) and groups (ACL‐R vs. controls) during the penultimate and final foot contacts.

**Results:**

No significant differences in COD completion time were found between limbs (*p* = 0.52, *d* = 0.22) or groups (*p* = 0.65, *d* = 0.51). However, during the penultimate foot contact, the involved limb exhibited greater ankle dorsiflexion compared to the uninvolved and controls from 48% to 100% of stance (*p* = 0.002, *d* = 0.94–1.86), with lower vertical force production (*p* > 0.05, *d* = 0.81–0.95). During the plant step, lower knee flexion angles were noted compared to the uninvolved limb and controls from 2% to 69% of stance (*p* = 0.011, *d* = 1.26–1.31).

**Conclusion:**

The findings suggest that soccer players with ACL‐R can restore COD completion time at the time to return to sport. However, they used compensatory movement strategies on the involved side to achieve similar performance, and this must be considered from a rehabilitation standpoint.

**Level of Evidence:**

Level III.

**Abbreviations**
ACL: anterior cruciate ligament
ACL‐R: anterior cruciate ligament reconstruction
CG: control group
COD: change of direction
IMU: inertial measurement unit
LSI: limb symmetry index
SD: standard deviation
SPM: statistical parametric mapping

## INTRODUCTION

Side‐step cutting is the most common change of direction (COD) manoeuvre in multidirectional sports like soccer [4]. This action is typically performed by players to create space for an opponent to receive a pass, or to penetrate a defensive line when attempting to initiate a scoring opportunity [33]. Despite its importance for successful performance during match‐play, side‐step cutting has been identified as a mechanism of non‐contact anterior cruciate ligament (ACL) injury [5, 37, 42]. This is mainly due to technical execution and biomechanical factors [10, 18]. Assessments of COD mechanics are not routinely included as part of a return‐to‐sports testing battery; therefore, practically viable approaches are warranted to increase their utilization.

Laboratory‐based studies have shown athletes can restore COD task completion time during return‐to‐sport assessments, but differences in COD mechanics remain [24, 25, 38]. Residual biomechanical deficits between limbs (involved vs. uninvolved) and groups (ACL vs. health control) associated with ACL injury have been observed 9 months post‐surgery despite athletes completing a comprehensive rehabilitation programme [24, 25, 38] during 45° [24, 38] and 90° side‐step cutting [24, 25, 38]. When interpreting these data, it should be considered that while laboratory‐based methods are considered the gold standard, their ecological validity can be limited. Tests are usually completed in a confined space where athletes are not able to achieve high approach speed, and contact must be made with a force plate, which constrains the movement task. There is a considerable time requirement for set‐up, data collection and post‐processing, in addition to the need for complex analysis and expensive technical equipment.

It should also be noted that laboratory studies examining COD ability following ACL reconstruction (ACL‐R) have only assessed the final foot contact (i.e., cutting step) [24, 25, 38]. Research has shown that sharp cutting angles (i.e., >60°) require greater reductions in velocity to change the athletes' state of momentum [17, 19]. To achieve a sufficient velocity reduction during a 90° side‐step cutting task, athletes must decelerate over multiple steps prior to the cutting step [14]. Accordingly, the penultimate foot contact plays a significant role in reducing velocity in preparation for optimal movement mechanics during the cutting step [23]. Examining the deceleration strategy is key to more comprehensively determining an athlete's COD ability. Currently, there is a paucity of available data to inform clinicians of the mechanics used by athletes during the penultimate and plant steps when returning to sport following ACL‐R.

Given these limitations, objective and practically viable measures to quantify movement strategies post‐ACL‐R are required to inform patients' readiness to return to sport. Wearable technology has emerged as a viable alternative for assessing movement mechanics directly on the pitch, preserving the athlete‐environment relationship. Marques et al. [31] conducted a literature synthesis and reported that wearable technology could consistently identify between‐limb (involved vs. uninvolved) and group (ACL vs. healthy controls) differences in kinetic and kinematic variables during functional tasks (e.g., walking, running and jumping/landing). However, the authors highlighted that the assessment of tasks relating to COD is sparse. Recent data indicate inertial measurement units (IMUs) provide valid estimates of lower extremity joint kinematics in the sagittal plane during COD tasks [20]. Similarly, insole sensors have been previously used with ACL reconstructed athletes during running and hop testing to estimate ground reaction force, but not with COD [36, 40]. Further research is required to elucidate if kinetics and kinematics measured using wearable technology can identify aberrant movement mechanics during COD tasks in athletes following ACL‐R.

To address these gaps identified in the literature, our objective was to bridge the gap between the lab and field by examining side‐step cutting performance and mechanics using wearable technology in athletes with a history of ACL‐R and healthy matched controls. We hypothesized that ACL‐reconstructed athletes would achieve similar COD task completion times to healthy‐matched controls but between‐limb and group differences in kinetics and kinematics, suggesting maladaptive movement strategies.

## MATERIALS AND METHODS

### Participants

Twenty‐six male professional soccer players (22.7 ± 3.7 years, height: 177.8 ± 5.1 cm: body mass: 69.4 ± 8.5 kg) participating in the Qatar Star League, which is the top professional tier in Qatar comprising both Qatari and international players including domestic competition and participation in the Asia Football Confederation Champions League were involved in this study. Ten of these players had a history of ACL injury and elected for surgical reconstruction (ACL‐R group, bone‐patella‐tendon bone graft = 60%, semitendinosus and gracilis hamstring tendon graft = 40%), and then underwent an intensive, supervised rehabilitation programme at the same Sports Medicine Hospital [27]. Assessments were completed at the time of return to sport (9.4 ± 2.03 months) after passing specific criteria, including (1) completion of a sport‐specific on‐field rehabilitation phase; (2) isokinetic quadriceps and hamstring strength limb symmetry index (LSI) > 90%; and (3) countermovement jump (jump height, eccentric and concentric impulse) and drop jump (jump height, reactive strength index, eccentric and concentric impulse) LSI > 90%. The remaining players were free from lower limb injury during the study, did not have a history of ACL injury/surgery, and were assigned as the control group (CG; *n* = 16). Each participant provided informed consent prior to participation.

### Experimental design

A within and between subject' cross‐sectional design was used to compare completion times, kinetics, and kinematics between limbs (involved vs. uninvolved) and groups (ACL‐R vs. control) during a COD task involving a 90° cut. Assessments were conducted on a temperature‐controlled indoor soccer pitch with artificial grass (3G system; type: monofilament; material: polypropylene, height: 40 mm, weight: 2 kg/m^2^; Direct Artificial Grass) with styrene‐butadiene rubber and quartz sand infill (infill characteristics installed following the manufacturer's guidelines) using their own soccer boots. A standardized warm‐up was completed prior, involving: (1) 3 mins of low‐intensity running to elevate heart rate followed by A‐ and B‐skipping exercises; (2) joint mobility and activation exercises (e.g., bilateral and unilateral squatting, walking lunges, bilateral and unilateral countermovement jumps); (3) four linear acceleration and deceleration efforts; (4) six familiarization trials (four submaximal trials with two efforts for each side followed by two maximal trials with one effort for each side).

### Procedures

#### COD testing

**Figure 1:**
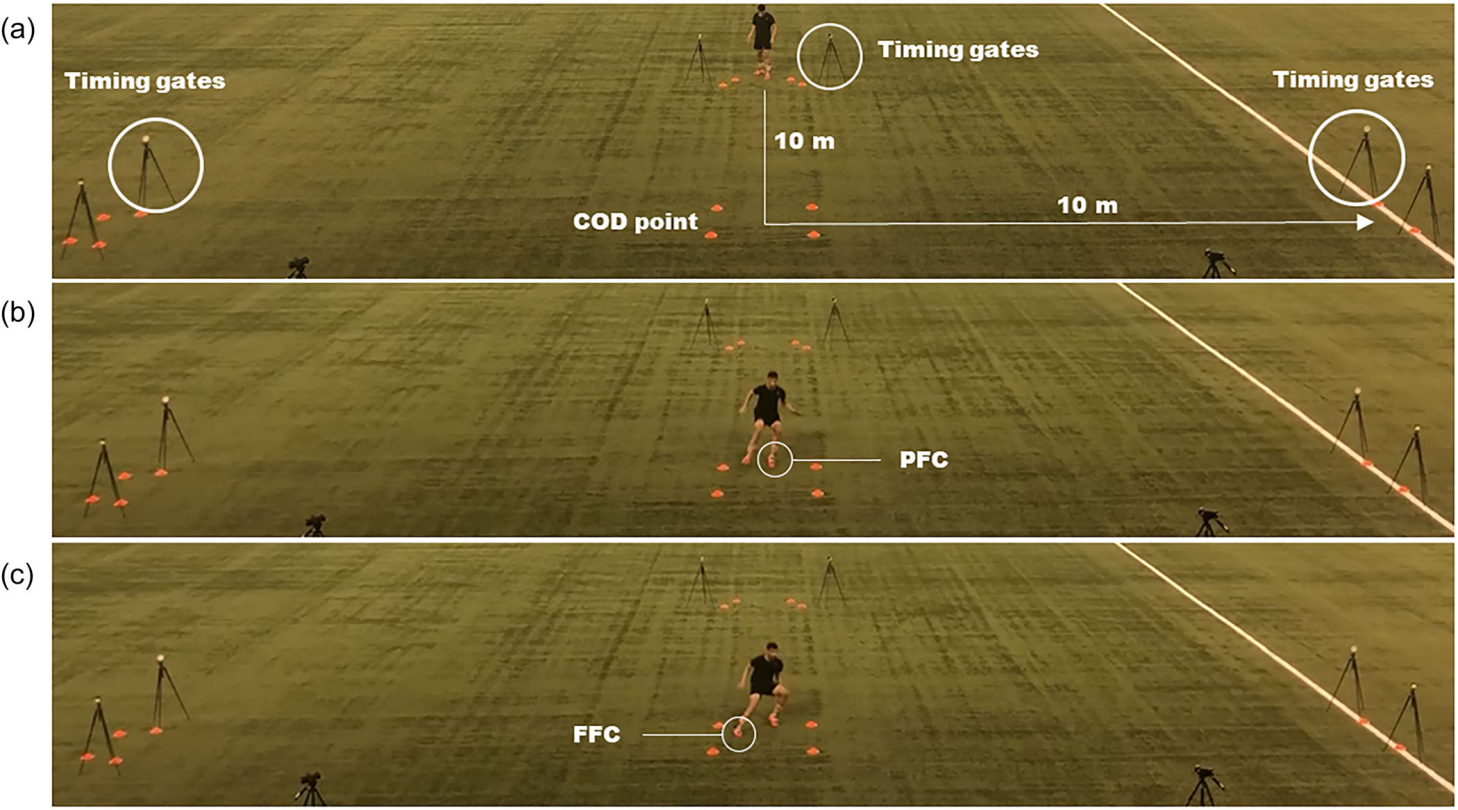
(a) Overview of the change of direction (COD) testing and placement of the timing gates to measure COD time; (b) Penultimate foot contact (PFC) to measure braking strategies; (c) Final foot contact (FFC) to measure COD strategy.

Each participant was required to perform a 20‐m ‘L‐shaped’ run (10 m linear sprint, 90° COD, followed by a second 10 m linear sprint) (Figure 1a). In total, six acceptable efforts were performed (three randomly allocated efforts for each side) interspersed by 2 min of passive recovery. The time to complete the task was measured through timing gates (Polifemo Microgate) positioned at the initial (0 m) and at the final (20 m) line of the task. The average value of the three trials performed for each side was selected for analysis.

#### Kinetic and kinematic measures

**Figure 2:**
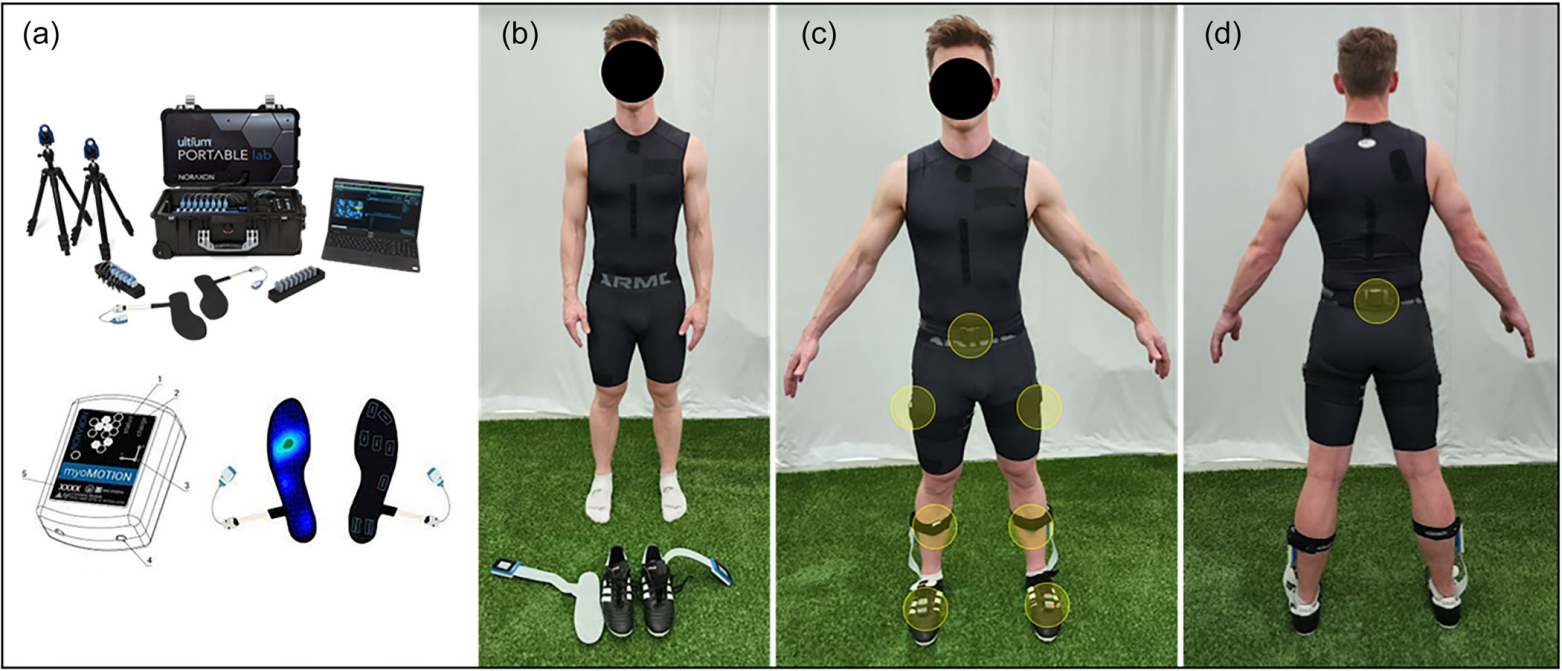
(a) Noraxon Portable Lab, inertial measurement unit (IMU) and insole sensors utilized during the study; (b) insole sensor placement; (c) IMU sensor placement (frontal view); (d) IMU sensor placement (posterior view).

Three‐dimensional kinematics, including hip, knee and ankle flexion angles, were measured with IMUs (Noraxon myoMOTION™ System Scottsdale), sampling at 200Hz. Recent data suggest these IMUs offer a valid cross‐correlation (XCORR) of lower extremity joint kinematics in the sagittal plane during COD tasks when compared to the gold‐standard 3D motion capture system, with XCORR values of 0.98 for the hip, 0.94 for the knee and 0.88 for the ankle [20]. The sensors were located on the pelvis (at the sacrum), and bilaterally on the lateral thighs (distal half where there is less muscle mass), shanks (anterior and slightly medial on the tibia) and midfoot (Figure 2c,d), in accordance with the rigid body model used in the Noraxon MR3 software (Noraxon myoMOTION™ System Scottsdale). Velcro straps and tape were used to fix the sensors. Preceding each COD trial, we performed a functional walking calibration procedure to ensure precise kinematic measurement as per manufacturer guidelines. The calibration process commenced with subjects assuming a static stance for 3 s, maintaining their arms at rest by their side and positioning their feet hip to shoulder width apart. Subsequently, subjects performed a 5 m walking trial at a self‐selected pace, including a 180° left turn, a return to the initial starting point, a resumption of the stationary posture for an additional 3 s, and then the calibration procedure was complete [43]. Joint and individual sensor orientation angles and angular velocities were recorded and further processed using the Noraxon MR3 software.

Medilogic® (T&T medilogic Medizintechnik GmbH) insoles were placed inside the soccer shoes (Figure 2b) and used to measure the estimated vertical force via the pressure distribution on the plantar surface of the feet during the COD task. Insole sensors have been previously used to identify asymmetry in ACL‐reconstructed athletes [36, 40]. Impulse and peak impact force symmetry measured using a force insole has been indicated as a significant predictor of kinetic knee symmetry measured using motion analysis and embedded tri‐axial force plates explaining 42%–61% of the variance [26]. There was also good agreement (ICCs = 0.742–0.862) between predicted and actual knee kinetic symmetry [26]. Each insole consists of 130 8‐bit sensors, each designed to withhold a maximum pressure of 64 N/cm^2^. To ensure correct pressure data, a zero‐load measurement was recorded for each insole prior to testing by consecutively lifting the feet off the floor. The pressure data were recorded at a frequency of 200 Hz and collected synchronously with the kinematic data using MR3 software. The estimated vertical force was derived from the sum of the total pressure distribution. All kinetic and kinematic variables were examined during the penultimate and final foot contact of the COD task to determine braking and COD strategies, respectively (Figure 1b,c).

The study was approved by the Anti‐Doping Laboratory, Doha, Qatar (Institutional Review Board: E202010016).

### Statistical analysis

A post hoc power calculation using G*Power (v3.1) was performed for the primary outcome measure (knee flexion angle) in this study. Based on a predefined meaningful difference of 7°, a standard deviation of 8°, a two‐sided *α* of 0.05, and a statistical power of 80%, the power calculation suggested that with 21 injured and 21 healthy athletes (total = 42 participants), the study is appropriately powered for between‐group comparisons. For within‐group comparisons (injured vs. non‐injured limb), a paired‐sample approach was considered, accounting for within‐subject correlation. If a higher statistical power of 90% is required, the total sample size would need to increase to 28 participants per group (56 total) to detect a statistical difference.

Descriptive statistics (mean ± SD) were calculated for the characteristics of the participants and the reported COD variables. All data were initially screened for veracity, including frequency histograms, *Q*–*Q* plots, and estimates of normality for the aggregated and group data. Subsequently, a mixed model analysis was conducted with the leg (involved, uninvolved and control) as a fixed factor and participants as random factors for each of the dependent variables. Where there were any significant differences identified (after Tukey HSD post hoc adjustment for multiple comparisons), models were checked for veracity by examining the distributions of the residuals (frequency histograms, *Q*–*Q* plots and Anderson–Darling tests of normality). Analyses were conducted in JMP software (JMP®, Version 17, SAS Institute Inc.). The level of significance was set at *p* < 0.05. Cohen's *d* effect sizes (ESs) were calculated using the pooled weighted standard deviation [21]. The magnitude of these differences was classified as follows: 0.2, 0.5 and 0.8 for small, moderate, and large effect sizes, respectively [7]. Statistical parametric mapping (SPM) analysis of the entire angle‐time and force‐time waveforms was conducted using SPM1D (Version 0.4.23) package in Python, from initial contact to toe‐off, during the penultimate and final foot contact of the COD task.

There were no significant differences in COD time, kinetics, or kinematics between the dominant and non‐dominant limbs for the CG (Table 1). Therefore, we used only one (randomly selected) limb from each control participant to conduct the between‐group analysis.

**Table 1:** Mean (±SD) for change of direction (COD) time, kinetics and kinematics for the dominant (DOM) and non‐dominant (NDOM) limbs analyzed during the penultimate and final foot contact of the COD task performed by the control group.

Variables assessed	DOM	NDOM	DOM‐NDOM		
			Difference (95% CI)	*p* value	*d*
COD time (s)	3.88 (±0.16)	3.91 (±0.13)	−0.04 (−0.16 to 0.09)	0.87	0.24
Penultimate foot contact					
Ankle dorsiflexion (°)	−6.9 (±9.11)	−6.5 (±6.91)	−0.42 (−8.12 to 7.26)	0.99	0.05
Knee flexion (°)	93.3 (±9.42)	97.8 (±8.49)	−4.47 (−13.16 to 4.20)	0.52	0.50
Hip flexion (°)	58.7 (±11.55)	62.2 (±9.29)	−3.52 (−12.76 to 5.71)	0.74	0.34
Vertical force (N/Kg)	1.4 (±0.52)	1.4 (±0.52)	0.02 (−0.39 to 0.42)	0.99	0.03
Final foot contact					
Ankle dorsiflexion (°)	20.8 (±8.88)	21.5 (±9.00)	−0.69 (−9.19 to 7.79)	0.99	0.08
Knee flexion (°)	48.8 (±6.98)	51.8 (±5.98)	−2.94 (−9.49 to 3.61)	0.63	0.45
Hip flexion (°)	35.0 (±8.00)	38.3 (±6.96)	−3.30 (−10.13 to 3.52)	0.57	0.44
Vertical force (N/Kg)	1.9 (±0.39)	1.8 (±0.53)	0.01 (−0.20 to 0.17)	0.88	0.03

Abbreviations: CI, confidence interval; d, effect size; SD, standard deviation.

## RESULTS

### Performance analysis—COD time

No significant differences in COD time were observed between the limbs and groups (Table 2).

**Table 2:** Mean (±SD) for change of direction (COD) time for the involved and the uninvolved limb and controls.

Variables	Involved	Uninvolved	Controls	Involved‐Uninvolved			Controls‐Involved			Controls‐Uninvolved		
Assessed				Difference (95% CI)	*p* value	*d*	Difference (95% CI)	*p* value	*d*	Difference (95% CI)	*p* value	*d*
COD time (s)	3.95 (±0.14)	3.98 (±0.14)	3.88 (±0.16)	−0.03 (−0.10 to 0.03)	0.52	0.22	−0.06 (−0.21 to 0.10)	0.65	0.51	−0.09 (−0.26 to 0.04)	0.37	0.71

Abbreviations: CI, confidence interval; d, effect size; SD, standard deviation.

### Biomechanical analysis—Penultimate foot contact

#### Ankle dorsiflexion angle

**Figure 3:**
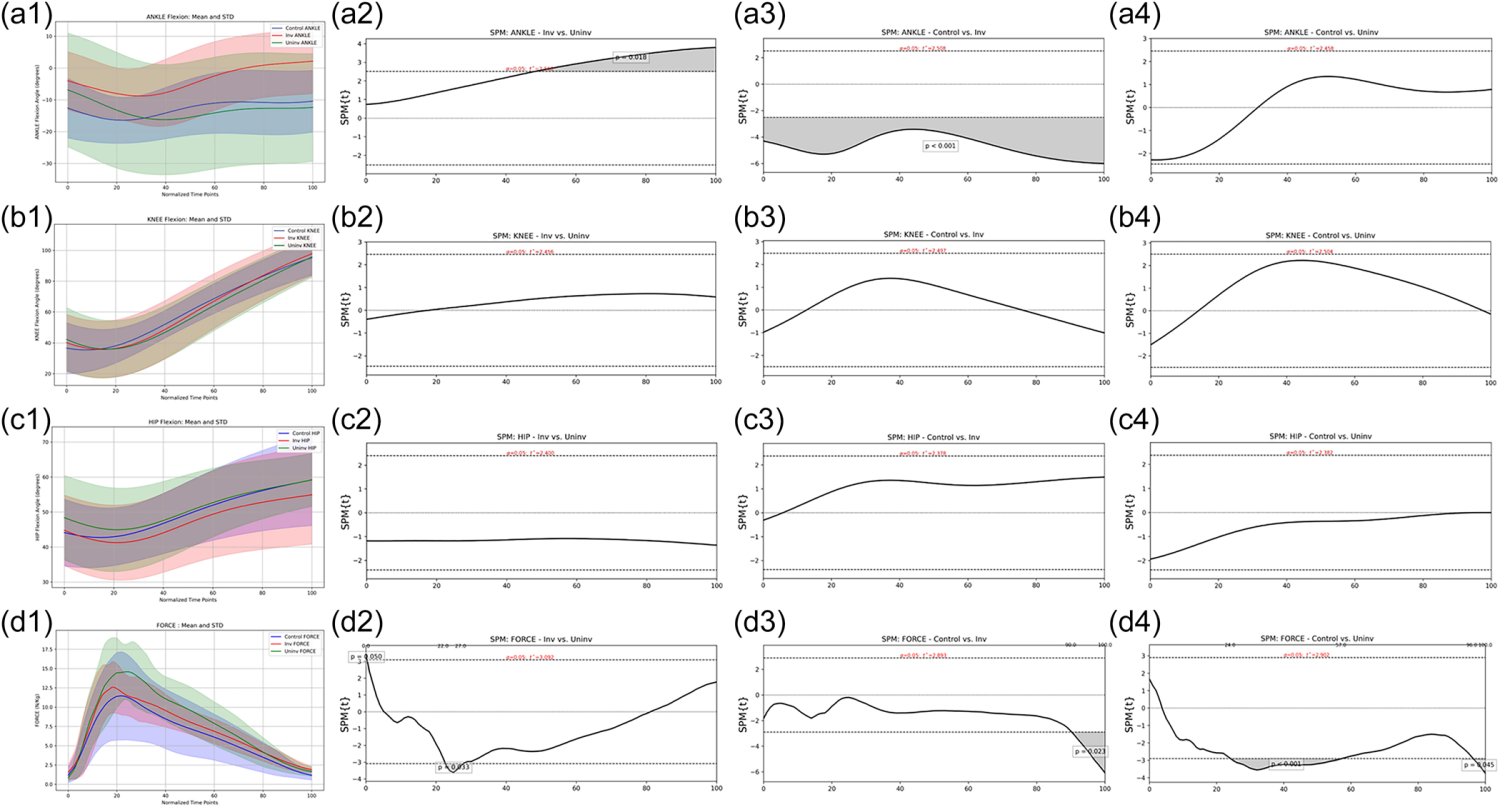
SPM analysis of the penultimate foot contact (from initial contact to toe‐off). Sub‐Figure 1 shows the mean and standard deviations, while sub‐Figures 2–3 show the SPM analysis throughout the entire phase, with significant differences shaded grey. (a1) mean ankle dorsiflexion angle (solid line) and standard deviation (shadow area) between involved versus uninvolved versus controls; (a2) mean differences in ankle dorsiflexion angle between involved versus uninvolved; (a3) mean differences in ankle dorsiflexion angle between controls versus involved; (a4) mean differences in ankle dorsiflexion angle between controls versus uninvolved; (b1) mean knee flexion angle (solid line) and standard deviation (shadow area) between involved versus uninvolved versus controls; (b2) mean differences in knee flexion angle between involved versus uninvolved; (b3) mean differences in knee flexion angle between controls versus involved; (b4) mean differences in knee flexion angle between controls versus uninvolved; (c1) mean hip flexion angle (solid line) and standard deviation (shadow area) between involved versus uninvolved versus controls; (c2) mean differences in hip flexion angle between involved versus uninvolved; (c3) mean differences in hip flexion angle between controls versus involved; (c4) mean differences in hip flexion angle between controls versus uninvolved; (d1) mean vertical force (solid line) and standard deviation (shadow area) between involved versus uninvolved versus controls; (d2) mean differences in vertical force between involved versus uninvolved; (d3) mean differences in vertical force between controls versus involved; (d4) mean differences in vertical force between controls versus uninvolved. SPM, statistical parametric mapping.

Peak ankle dorsiflexion angle was significantly higher in the involved limb compared to the uninvolved and controls (Table 3). Similarly, SPM analysis revealed that the involved limb exhibited a significantly higher ankle dorsiflexion angle compared to the uninvolved side (*p* < 0.05) from 48% to 100% of the stance phase (Figure 3a2) and compared to the CG (*p *< 0.001) throughout the entire penultimate foot contact (Figure 3a3).

**Table 3:** Mean (±SD) for kinetic and kinematic variables for the involved and the uninvolved limb and controls analyzed during the penultimate foot contact of the change of direction (COD) task.

Variables	Involved	Uninvolved	Controls	Involved‐Uninvolved			Controls‐Involved			Controls‐Uninvolved		
Assessed				Difference (95% CI)	*p* value	*d*	Difference (95% CI)	*p* value	*d*	Difference (95% CI)	*p* value	*d*
Ankle dorsiflexion (°)	6.8 (±5.22)	−2.5 (±13.96)	−6.4 (±9.65)	9.38 (0.95–17.80)	0.026^a^	0.98^b^	−13.52 (−22.10 to −4.94)	0.001^a^	1.78^b^	−4.14 (−12.72 to 4.43)	0.46	0.33
Knee flexion (°)	99.0 (±11.31)	95.6 (±10.76)	92.8 (±9.54)	3.39 (−4.94 to 11.74)	0.58	0.31	−4.48 (−14.23 to 5.27)	0.50	0.60	−1.08 (−10.83 to 8.67)	0.95	0.28
Hip flexion (°)	56.9 (±13.71)	59.9 (±7.92)	57.6 (±11.70)	−2.93 (−8.52 to 2.66)	0.41	0.27	2.14 (−8.48 to 12.77)	0.87	0.06	−0.78 (−11.41 to 9.84)	0.98	0.23
Vertical force (N/Kg)	1.5 (±0.34)	1.8 (±0.34)	1.4 (±0.51)	−0.28 (−057 to 0.01)	0.06	0.82^b^	−0.10 (−0.56 to 0.35)	0.84	0.16	−0.38 (−0.84 to 0.07)	0.11	0.81^b^

Abbreviations: CI, confidence interval; d, effect size; SD, standard deviation.

^a^ Significant difference.

^b^ Large effect size.

#### Knee flexion angle

No significant differences in peak knee flexion angle were observed between limbs and groups (Table 3). Similarly, SPM analysis revealed no significant differences in knee flexion angles between limbs and between groups during the penultimate foot contact (Figure 3b1–b4).

#### Hip flexion angle

No significant differences in peak hip flexion angle were observed between limbs and groups (Table 3). Similarly, SPM analysis revealed no significant differences in hip flexion angles between limbs and between groups during the penultimate foot contact (Figure 3c1–c4).

#### Vertical force

Large effect sizes were observed in peak vertical force between limbs and groups (Table 3). SPM analysis revealed that the involved limb exhibited a significantly lower vertical force compared to the uninvolved side (*p* < 0.05) from 22% to 27% of the stance phase (Figure 3d2) but higher vertical force compared to the CG (*p* < 0.05) from 90% to 100% of the stance phase (Figure 3d3). Similarly, the uninvolved limb exhibited a significantly higher vertical force compared to the CG (*p* < 0.05) from 24% to 57% and from 96% to 100% of the stance phase (Figure 3d4).

### Biomechanical analysis—Final foot contact

#### Ankle dorsiflexion angle

**Figure 4:**
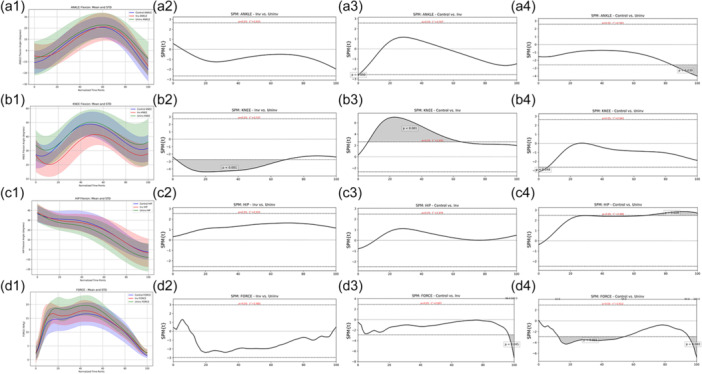
SPM analysis of the final foot contact (from initial contact to toe‐off). Sub‐Figure 1 shows the mean and standard deviations, while sub‐Figures 2–3 show the SPM analysis throughout the entire phase, with significant differences shaded grey. (a1) mean ankle dorsiflexion angle (solid line) and standard deviation (shadow area) between involved versus uninvolved versus controls; (a2) mean differences in ankle dorsiflexion angle between involved versus uninvolved; (a3) mean differences in ankle dorsiflexion angle between controls versus involved; (a4) mean differences in ankle dorsiflexion angle between controls versus uninvolved; (b1) mean knee flexion angle (solid line) and standard deviation (shadow area) between involved versus uninvolved versus controls; (b2) mean differences in knee flexion angle between involved versus uninvolved; (b3) mean differences in knee flexion angle between controls versus involved; (b4) mean differences in knee flexion angle between controls versus uninvolved; (c1) mean hip flexion angle (solid line) and standard deviation (shadow area) between involved versus uninvolved versus controls; (c2) mean differences in hip flexion angle between involved versus uninvolved; (c3) mean differences in hip flexion angle between controls versus involved; (c4) mean differences in hip flexion angle between controls versus uninvolved; (d1) mean vertical force (solid line) and standard deviation (shadow area) between involved versus uninvolved versus controls; (d2) mean differences in vertical force between involved versus uninvolved; (d3) mean differences in vertical force between controls versus involved; (d4) mean differences in vertical force between controls versus uninvolved. SPM, statistical parametric mapping.

Peak ankle dorsiflexion angle was significantly lower in the involved versus uninvolved limb, while large effect sizes were observed between groups (Table 4). SPM analysis revealed that the uninvolved limb exhibited a significantly higher (*p* < 0.05) ankle dorsiflexion angle from 84% to 100% of the stance phase when compared to the controls (Figure 4a4). No significant differences were observed between limbs in the ACL‐R group and between the involved limb and controls (Figure 4a2,a3).

**Table 4:** Mean (±SD) for kinematic variables for the involved and the uninvolved limb and controls analyzed during the final foot contact of the change of direction (COD) task.

Variables	Involved	Uninvolved	Controls	Involved‐Uninvolved			Controls‐Involved			Controls‐Uninvolved		
Assessed				Difference (95% CI)	*p* **value**	*d*	Difference (95% CI)	*p* **value**	*d*	Difference (95% CI)	*p* **value**	*d*
Ankle dorsiflexion (°)	19.6 (±11.63)	27.3 (±8.24)	20.0 (±8.87)	−7.67 (−14.73 to −0.60)	0.031^a^	0.86^b^	1.12 (−7.97 to 10.21)	0.95	0.04	−6.55 (−15.64 to 2.54)	0.19	0.84^b^
Knee flexion (°)	42.9 (±8.08)	57.5 (±16.76)	50.3 (±10.1)	−14.55 (−24.60 to −4.49)	0.003^a^	1.17^b^	7.38 (−1.43 to 16.20)	0.11	0.81^b^	−7.16 (−15.98 to 1.65)	0.12	0.54
Hip flexion (°)	38.9 (±7.81)	38.6 (±8.75)	35.2 (±8.00)	0.39 (−4.93 to 5.72)	0.98	0.05	−1.96 (−9.59 to 5.65)	0.80	0.50	−1.57 (−9.20 to 6.05)	0.86	0.40
Vertical force (N/Kg)	2.0 (±0.35)	2.1 (±0.50)	1.9 (±0.39)	−0.11 (−0.41 to 0.19)	0.64	0.26	0.11 (−0.56 to 0.34)	0.82	0.29	−0.22 (−0.67 to 0.22)	0.45	0.49

Abbreviations: CI, confidence interval; d, effect size; SD, standard deviation.

^a^ Significant difference.

^b^ Large effect size.

#### Knee flexion angle

Peak knee flexion angle was significantly lower in the involved limb compared with the uninvolved and controls (Table 4). SPM analysis revealed that the involved limb exhibited a significantly lower (*p* < 0.01) knee flexion angle compared to the uninvolved from 2% to 69% of the stance phase (Figure 4b2). Similarly, the involved limb exhibited a significantly lower (*p* < 0.01) knee flexion angle compared to the CG from 6% to 64% of the stance phase (Figure 4b3). The uninvolved limb exhibited a significantly higher (*p* < 0.05) knee flexion angle compared to the CG from 0% to 5% of the stance phase (Figure 4b4).

#### Hip flexion angle

No significant differences in peak hip flexion angle were observed between limbs and between groups (Table 4). SPM analysis revealed that the uninvolved limb exhibited a significantly lower (*p* < 0.05) hip flexion angle compared to the CG from 67% to 100% of the stance phase (Figure 4c4). No significant differences in hip flexion angle were observed between limbs (ACL‐R group) and between the involved limb and controls during the final foot contact (Figure 4c2,c3).

#### Vertical force

No significant differences in peak vertical force were observed between limbs and groups (Table 4). SPM analysis revealed that the involved limb exhibited a significantly higher (*p* < 0.05) vertical force compared to the CG from 96% to 100% of the stance phase (Figure 4d3). Similarly, the uninvolved limb displayed significantly higher (*p* < 0.05) vertical force compared to controls from 12% to 54% and from 94% to 100% of the stance phase (Figure 4d4). No significant difference was observed in vertical force between limbs during final foot contact (Figure 4d2).

## DISCUSSION

The objective of the present study was to use wearable technology to examine side‐step cutting performance and mechanics in athletes with a history of ACL‐R. We also made comparisons to healthy matched controls. Our findings indicate that task completion time was comparable; however, biomechanical differences in ankle and knee kinematics and vertical force production exist between limbs (involved vs. uninvolved) and groups (ACL‐R vs. CG) at the time of return to sport after ACL‐R. In line with previous research [24, 25, 38], our findings indicate that using completion time solely to measure COD is not sufficient to identify differences in movement mechanics adopted by athletes. The biomechanical differences observed should be considered in the design of on‐field rehabilitation programmes and during return to sport monitoring.

### Performance—COD time

Time to complete the side‐step cutting task was not significantly different between the involved and the uninvolved limb and controls. This suggests that soccer players with ACL‐R were able to restore COD time prior to return to sport after ACL‐R. However, different strategies were identified, indicating the presence of compensatory movement patterns. Previous laboratory‐based research has reported residual biomechanical deficits between the involved and uninvolved limb despite no differences in COD time in male team‐sport athletes at 9 months after ACL reconstruction [24]. However, COD time has been used previously as a measure of rehabilitation status after ACL‐R [28, 34], but this metric alone may not be suitable to determine readiness to return to sport. Cumulatively, this suggests the need to examine potential deficits in lower extremity biomechanics, which may contribute to a greater risk of re‐injury [24, 25, 38].

### Biomechanical differences—Penultimate foot contact

Kinematic alterations occurred mainly at the ankle joint, with significantly higher dorsiflexion on the involved limb compared with the controls and uninvolved limb. This movement strategy was adopted by the involved limb during the entire stance phase of the penultimate foot contact compared with controls (0%–100%) and from 48% to 100% of the stance phase when compared to the uninvolved limb. A reduction in vertical force production during the penultimate foot contact compared to the uninvolved side was also present, particularly in the early stance phase. This may suggest an offloading mechanism to reduce the force production requirement on the reconstructed limb. During rapid deceleration, a high internal knee extensor moment is required to control and attenuate forces across relevant knee joint flexion ranges, particularly during the early phases of braking when high impact forces and loading rates may be experienced [23, 39]. This strategy might be the result of lower strength in the quadriceps muscles, which can persist for several years after ACL‐R [6, 32].

Research has shown that faster COD performance times are associated with greater horizontal braking forces in the penultimate foot contact prior to a change the direction [10]. This braking strategy reduces horizontal momentum prior to the final foot contact to facilitate more effective weight acceptance and push‐off propulsive forces during the final foot contact, contributing to faster COD performance [10]. We observed moderate‐large effect sizes for COD completion time between the uninvolved limb and controls. This might be associated with the reduction in vertical force produced by the involved limb during the penultimate foot contact. It is plausible that this strategy induced more mechanical work for the uninvolved limb to decelerate the horizontal momentum during the cutting step, increasing the time spent changing direction and subsequently the overall task completion time. Cumulatively, these data indicate that the restoration of quadriceps strength and rate of force production should be a key focus of reconditioning programmes following surgery.

When interpreting the kinematic data, it should be considered that the ankle dorsiflexion angle displayed the largest variability. This may be associated with the setup of the COD task employed in our study. We have used a 10 m entry distance, which allows athletes to achieve 72% of their maximal velocity prior to decelerating [16]. Previous lab‐based studies used 5 m only [24, 25, 38], during which only 54% of maximal velocity is reached [16]. Decelerating from higher entry velocities is more challenging than from lower velocities because it requires greater braking forces to reduce the increased momentum [16]. Athletes were also not constrained to place their feet over embedded force plates (as required during lab‐based protocols). This affords the opportunity to employ different deceleration techniques, particularly at the ankle joint, which plays an important role in shock attenuation prior to performing the COD task. These data support the notion that targeted training to develop the force production abilities of the plantar flexors is also an important component of a comprehensive return to performance programme.

### Biomechanical differences—Final foot contact

The knee joint displayed the largest kinematic alterations during the plant step, with less knee flexion on the involved compared to the uninvolved limb and controls throughout most of the stance phase (0%–70%). ACL‐reconstructed players also displayed reduced ankle dorsiflexion compared to the uninvolved limb, supporting the idea that players with ACL‐R adopted a more extended lower extremity position (i.e., less knee and ankle dorsiflexion angles) to execute the COD task on the involved side. This strategy may have been adopted to reduce the internal knee extensor moment required to act against the large external knee flexion moment when braking to reduce the horizontal momentum of the centre of mass [22].

This extended lower extremity position (i.e., less knee flexion angle), in combination with other technical aspects (greater lateral foot placement, final foot braking forces in short ground contact times and high centre of mass velocity), has been associated with faster side‐step performance time [15]. However, adopting these mechanics while changing direction can increase ACL strain and has been associated with the mechanism of ACL injury [1]. This is attributed to the greater anterior tibial shear force [29] and unopposed action of the quadriceps in this position thus exposing the involved limb to a greater risk of re‐injury [22].

Previous research has reported shallower knee flexion angles on the involved limb during a side‐step cutting task at the time to return to sport in team‐sport athletes following ACL‐R [24]. Reduced knee flexion angle has also been reported during the stance phase of running at different time points after ACL‐R and has been related to reductions in quadriceps strength or an avoidance strategy [2, 35]. The more joints are flexed upon initial contact with the ground, the more the energy is absorbed and the less the impact is transferred to the knee [1]. The reduced ability to absorb load on the involved limb (i.e., reduced knee flexion angle) and decrease ACL strain (i.e., increased knee flexion angle) while changing direction may influence the risk of re‐injury [8, 24] and highlights a potential area to be targeted during the rehabilitation process. This should involve both physical capacity development and technical skill training in which athletes are coached effectively to adopt mechanics that optimize performance and reduce injury risk, as shown in previous research [13].

The hip flexion angle was also moderately greater on the involved side, suggesting a compensatory movement strategy to assist with a potential reduction in internal knee extensor moment capacity. The hamstring and gluteal muscle groups are responsible for preventing anterior tibial displacement and a dynamic valgus collapse of the knee, respectively [9]. Increasing hip flexion changes the moment arm and lengthens the hamstring muscles [9]. Adopting a movement strategy where the ankle and the knee are close to full extension in addition to an increased hip flexion angle may place the ACL reconstructed players in a more vulnerable position to execute the COD task during the final foot contact, increasing the risk of re‐injury.

### Practical implications

We observed that ACL‐reconstructed athletes were able to restore COD completion time, but this was accompanied by residual biomechanical deficits. The extended lower extremity position we observed has been associated with the mechanism of ACL injury. When the knee is near full extension, the ACL has a greater elevation angle, so the ligament is more perpendicular to the tibial plateau line [30]. This change in orientation influences the load placed on the ACL and its ability to sustain elastic deformation without injury [3]. As the knee progresses into extension, the ACL elevation angle is maximized. Under this configuration, the anterior tibial shear force generated by the quadriceps/patellar tendon and imparted to ACL is increasingly shear in nature [3]. At lower knee flexion angles, the quadriceps exert a higher anteriorly directed force that is poorly counteracted by both the ACL and hamstrings [44].

Understanding how braking and deceleration (i.e., penultimate foot contact) influence cutting strategies (i.e., final foot contact) is essential for practitioners currently working with ACL‐reconstructed athletes who intend to return to cutting and pivoting sports like soccer. Our findings and previous laboratory‐based research [24, 25, 38] suggest that focus should be placed on COD mechanics, rather than solely on COD performance time during rehabilitation. Our results suggest that wearable technology is a suitable tool to monitor rehabilitation status as it is sensitive to identifying residual biomechanical deficits. This may support practitioners when profiling COD mechanics, which in turn can aid the return‐to‐sports decision‐making process. In addition, the practical utility of this tool may permit the use of wearable technology during training sessions to provide feedback on COD mechanics, ensuring test‐training integration.

### Limitations and future directions

When interpreting the findings of our study, readers should be aware of some limitations. First, we did not examine frontal and transverse plane motion. This is due to poor agreement and high variability reported by previous research investigating the differences between IMU sensors and 3D motion capture systems to measure COD mechanics [20]. Future research is required to determine whether IMU sensors are valid and reliable for measuring frontal and transverse plane motion during COD tasks.

Readers should also be aware that although pressure insoles have been shown to be reliable for assessing vertical ground reaction forces in field situations, they lack the ability to measure horizontal forces, which is an important determinant factor for faster cutting manoeuvres [12]. An alternative could be using embedded force plates. However, this method may alter the athlete's ‘normal’ movement pattern, as they are required to make contact on the force plate. It was not our objective to constrain the athletes, but instead, to offer them the opportunity to use their preferred deceleration and cutting strategy.

Another point to consider is that we assessed side‐step cutting only and results cannot be transferred to other COD tasks as different techniques and movement strategies are adopted [11]. Similarly, while we standardized the surface used, players completed the assessment in their own boots. Different outsoles can affect the traction properties and potentially alter mechanics [41]. Future research should examine if wearable technology is effective in profiling differences in COD mechanics across different cutting/turning angles and if shoe outsole type significantly affects task execution.

Finally, we were only able to recruit a small sample size, and this may have impacted the ability of our statistical model to accurately assess the distribution of residuals for the knee joint angle. We suggest our data should be considered preliminary or pilot and should not extrapolate to the wider population. However, we observed large effect sizes, and the differences observed in SPM analysis appear to confirm that meaningful kinematic alterations occurred at the knee joint on the involved limb, which warrants further investigation. Future studies should aim to include larger cohorts both with and without ACL‐R to determine normative values and thresholds associated with injury risk.

## CONCLUSION

This is the first study using wearable technology to examine on‐field COD performance and movement mechanics in professional soccer players with a history of ACL‐R at the time of return to sport. We observed biomechanical differences between limbs and groups were more pronounced than task completion time, specifically in vertical force and ankle and knee kinematics. This implies a compensatory movement strategy, which could be linked to future injury risk. Incorporating rehabilitation practices that focus on COD movement patterns may prove effective in restoring proper mechanics. We recommend that practitioners assess movement strategies during rehabilitation, instead of solely relying on task completion time, to obtain more objective data when making return‐to‐sport decisions. Our preliminary findings suggest that wearable technology can identify residual kinetic and kinematic deficits following ACL‐R and may provide a viable alternative to laboratory‐based protocols, helping bridge the gap between the lab and the field.

## AUTHOR CONTRIBUTIONS

The authors Joao Belleboni Marques, Vasileios Sideris and Paulo Roberto Pereira Santiago were involved in the conceptualization of the project. The data collection and formal analysis were performed by Joao Belleboni Marques, Vasileios Sideris and Rodney Whiteley and further reviewed and validated by Paul James Read and Matheus Machado Gomes. All authors commented, revised and approved the manuscript drafted by Joao Belleboni Marques.

## CONFLICT OF INTEREST STATEMENT

The authors declare no conflicts of interest.

## ETHICS STATEMENT

The study was approved by the Anti‐Doping Laboratory, Doha, Qatar (Institutional Review Board: E202010016). In accordance with ethical guidelines, informed consent was obtained from all participants involved in this study. The participants were provided with comprehensive information about the purpose of the research, the procedures involved, and any potential risks. They were also informed of their right to withdraw from the study at any time without consequence. All participants gave their written consent for their data to be used in this research and for any publication of the results, with the understanding that their personal identifiers would remain confidential.

## Data Availability

The data that support the findings of this study are available from the corresponding author upon request.
